# Regulation of Brain-Derived Neurotrophic Factor Exon IV Transcription through Calcium Responsive Elements in Cortical Neurons

**DOI:** 10.1371/journal.pone.0028441

**Published:** 2011-12-09

**Authors:** Fei Zheng, Xianju Zhou, Yongneng Luo, Hua Xiao, Gary Wayman, Hongbing Wang

**Affiliations:** 1 Department of Biochemistry and Molecular Biology, Michigan State University, East Lansing, Michigan, United States of America; 2 Department of Physiology, Michigan State University, East Lansing, Michigan, United States of America; 3 Cellular and Molecular Biology Program, Michigan State University, East Lansing, Michigan, United States of America; 4 Neuroscience Program, Michigan State University, East Lansing, Michigan, United States of America; 5 Department of Veterinary and Comparative Anatomy, Pharmacology and Physiology, Washington State University, Pullman, Washington, United States of America; Centre National de la Recherche Scientifique - University of Bordeaux, France

## Abstract

Activity-dependent transcription of brain-derived neurotrophic factor (BDNF) has been studied as an important model to elucidate the mechanisms underlying numerous aspects of neuroplasticity. It has been extensively emphasized that Ca^2+^ influx through different routes may have significantly different effects on BDNF transcription. Here, we examined the regulatory property of the major calcium responsive elements (CaRE) in BDNF promoter IV in cultured rat cortical neurons. BDNF promoter IV, as well as CaRE1 and CaRE3, was significantly activated by Ca^2+^ influx through L-type voltage-gated calcium channel (L-VGCC) or NMDA receptor (NMDAR). However, the L-VGCC- and NMDAR-mediated activation of CaRE was differentially regulated by different Ca^2+^-stimulated protein kinases. Specifically, PKA, CaMKI, and CaMKIV activity were required for L-VGCC-, but not NMDAR-mediated CaRE1 activation. CaMKI activity was required for NMDAR- but not L-VGCC-mediated CaRE3 activation. Surprisingly, the activation of CaRF, a previously identified transcription factor for CaRE1, was stimulated via L-VGCC but not NMDAR, and required MEK, PI3K and CaMKII activity. These results suggest a new working model that activity-dependent BDNF IV up-regulation may be coordinately mediated by CaRE1 and CaRE3 activity, which show different responses to Ca^2+^-stimulated kinases. Our data also explain how the individual *cis*-element in BDNF promoter is distinctively coupled to different Ca^2+^ routes.

## Introduction

Activity-dependent gene transcription is functionally relevant for animals to acquire information and adapt to their environments. One major molecule that transduces neuronal activity and regulates transcription is calcium. Ca^2+^ influx through voltage-gated and ligand-gated Ca^2+^ channels triggers the activation of multiple protein kinases, and subsequently regulates transcription factors and transcription initiation.

Previous studies on transcription factor CREB (cAMP responsive element binding protein) have revealed how transcription initiation can be tightly and specifically controlled by multiple signaling activities triggered by Ca^2+^ [1], [2], [3]. Specifically, multiple phosphorylation sites in CREB are regulated by calmodulin (CaM)-dependent protein kinases, cAMP-dependent protein kinase (PKA), and the Ras/Raf/MAPK/Rsk cascade. Further, lack of CREB-mediated transcription is implicated in mental disorders [4], [5], neurodegeneration [6], apoptosis during development [7], [8], and impaired synaptic plasticity [9].

One important gene target of CREB is brain-derived neurotrophic factor (BDNF). BDNF transcription is very responsive to neural activity. It is up-regulated by learning [10], [11], physical exercise [12], and kindling or kainite-induced seizures [13]. The induction of BDNF expression could theoretically exert further modification on synaptic functions, such as regulating dendritic spine density [14], [15], enhancing both pre-synaptic and post-synaptic functions [16], [17], and mediating long-term potentiation (LTP) and memory formation [18], [19].

Molecular studies have revealed that the BDNF gene consists of nine 5′ exons (from exon I to IXA) and a common 3′ encoding exon IX [20]. After transcription and splicing, one and only one 5′ exon is joined to exon IX, resulting in nine different BDNF mRNA forms, each of which contains one 5′ exon and the exon IX. In cultured cortical neurons, Ca^2+^ influx through L-type voltage-gated calcium channel (L-VGCC) and NMDA receptor (NMDAR) specifically stimulates the transcription of exon IV-containing BDNF mRNA or BDNF IV [21], [22], [23] (because of the recent discovery of new exons, exon IV was described as exon III in the earlier studies). The 1500 bp of the 5′ flanking sequence of exon IV (defined as promoter IV) confers the transcriptional activity that is regulated by Ca^2+^ stimulation [21]. Truncation and mutagenesis analysis have identified three calcium responsive elements, namely CaRE1, CaRE2, and CaRE3 [24], [25]. By using a yeast one-hybrid screening, transcription factors CaRF (Calcium responsive factor) and USF (upstream stimulatory factors) have been found to bind CaRE1 and CaRE2, respectively [24], [25]. *In vitro* and *in vivo* studies have demonstrated the binding of CREB to CaRE3 [21], [26]. Although it is well established that the activity-dependent BDNF transcription depends on Ca^2+^-stimulated protein kinases, how Ca^2+^ regulates the individual CaRE is not known. In addition, although Ca^2+^ influx through different routes (e.g. through L-VGCC and NMDAR) may have significantly different impacts on BDNF transcription [2], to our knowledge, how Ca^2+^ influx through L-VGCC and NMDAR could dictate different regulation of BDNF transcription remains obscure.

To investigate these important issues, we used pharmacological inhibition and dominant negative constructs of the major Ca^2+^-stimulated protein kinases to examine the regulation of BDNF exon IV transcription, promoter IV activity, and CaRE activity in cultured neurons. Our results suggest that the individual CaRE coordinates with each other to regulate promoter IV activity. Our data also demonstrated that the activity of CaRE was differentially regulated by Ca^2+^-stimulated protein kinases, and showed different regulatory properties in response to Ca^2+^ influx through L-VGCC and NMDAR.

## Materials and Methods

### Reagents

All chemical reagents were purchased from Sigma (St. Louis, MO), unless otherwise stated. LY294002 (a PI3K inhibitor), PD98059 (a MEK inhibitor), and H89 (a PKA inhibitor) were purchased from Calbiochem (Gibbstown, NJ). Oligonucleotides were synthesized by Integrated DNA Technologies (Coralville, IA). Cell culture and transfection reagents were from Invitrogen (Carlsbad, CA).

### Plasmids and reporter constructs

The 911 bp promoter IV region of BDNF was cloned from rat genomic DNA with the specific forward primer (5′-ATGCTCGAGAAGAGGCTGTGGCACCGTGC-3′) and the reverse primer (5′-CCCAAGCTTTCCCCAAGGTTCTAGACTC-3′). The fragment was inserted into the XhoI/HindIII site of pGL3-basic firefly luciferase reporter vector (Promega, Madison, WI) to generate PIV-Luc. Three copies of CaRE1, or CaRE2, or CaRE3 [24] sequence in BDNF promoter IV were cloned into pGL3-promoter firefly luciferase reporter vector, which contains a minimal SV40 promoter, to generate plasmids CaRE1-Luc, CaRE2-Luc and CaRE3-Luc. The CRE-Luc reporter plasmid was described previously [27], and was a gift from Dr. Daniel Storm (University of Washington). Gal4, Gal4-CaRF, Gal4-CREB, and UAS-Luciferase constructs [24] were generously provided by Dr. Michael Greenberg (Harvard University). The Renilla Luciferase reporter construct, TK-pRL (Renilla-luciferase), was from Dr. Richard Miksicek (Michigan State University). Expression plasmids of dominant negative PKA (dnPKA, which harbors mutations at the cAMP binding sites) [27], dominant negative MEK (dnMEK, which harbors a S222A mutation) [27], dominant negative PI3K (dnPI3K, p110ΔKIN) [28], dominant negative CaMKI (dnCaMKI, which harbors multiple mutations in the catalytic residues, CaMKK phosphorylation domain, and the autoinhibitory domain), dominant negative CaMKIV (dnCaMKIV, which harbors multiple mutations in the ATP binding sites and the autoinhibitory domain with a nuclear localization signal attached) [29], and CaMKIIN (which expresses a CaMKII inhibitory protein) [30] were described previously. Dominant negative calmodulin (pJPA7/rCaM-DEF1234A) [31] was from Dr. Philippe Marin (University of Montpellier, France). Plasmid expressing a constitutively active CREB (VP16-CREB, in which the first Gln-rich domain of CREB is replaced by the transactivation domain of Herpes simplex virus VP16) [32] was from Dr. Karl Obrietan (Ohio State University).

### Primary culture of cortical neurons

The Institutional Animal Care and Use Committee at Michigan State University have approved all the methods in this study. The approval ID is 11/10-182-00. As described previously [33], brain tissue was obtained from post-natal day 0 Sprague Dawley rats. Dorsal and frontal cortical regions were dissected and incubated in the digestion buffer containing 10 unit/ml papain (Worthington), 100 unit/ml DNase I (Roche), and 5 mg/ml cystine (Sigma) in Hibernate A (BrainBits LLC). After 30 min digestion and dispersion, neurons were seeded on poly-D-lysine (50 µg/ml, Sigma, St. Louis, MO)-coated plates at a density of 0.15 million cells/cm^2^. The culturing medium was Neurobasal A with B27 supplement, penicillin/streptomycin, and 0.5 mM glutamate.

### Neuronal stimulation

Ca^2+^ influx through L-VGCC was triggered by membrane depolarization with KCl (at 50 mM) treatment. Neurons were first pre-treated with APV (100 µM) and CNQX (40 µM) for 30 min before the addition of KCl. Ca^2+^ influx through NMDA receptor was triggered by NMDA (20 µM) and glycine (2 µM) treatment. Neurons were first pre-treated with nifedipine (10 µM) and CNQX (40 µM) for 30 min before adding NMDA/glycine. APV, nifedipine, and CNQX were used to block NMDAR, L-type voltage-gated calcium channel, and non-NMDA type glutamate receptors, respectively. To inhibit MEK, PI3K, PKA, and CaMKs, neurons were pre-treated for 30 min before stimulation with PD98059 (50 µM), LY294002 (30 µM), H89 (10 µM), and KN93 (5 µM), respectively. To stop transcription, a transcription inhibitor actinomycin D (ACD) was used at a concentration of 100 ng/ml. The stimulation or inhibition was done by adding the drugs directly into the culture medium.

### Real-time PCR

Neurons were treated on 14 days *in vitro* (DIV). After stimulation with KCl or NMDA, control and stimulated neurons were lysed. Total RNA was extracted with TRIzol (Invitrogen). RNA concentration was measured by NanoDrop 1000 (Thermo, Waltham, MA), and 0.5 µg RNA was reverse transcribed to cDNA with the SuperScript III kit (Invitrogen). Exon IV-containing BDNF (BDNF IV) mRNA and GAPDH mRNA were amplified by the iQ SYBR Green real-time PCR system (Bio-Rad, Hercules, CA), and analyzed with the 2^−ΔΔCt^ method by normalizing BDNF IV signal to GAPDH. The annealing temperature for both genes was 55°C. The primers were 5′-CTCCGCCATGCAATTTCCAC-3′ and 5′-GCCTTCATGCAACCGAAGTA-3′ for BDNF exon IV. Primers 5′-TCCATGACAACTTTGGCATTGTGG-3′ and 5′-GTTGCTGTTGAAGTCGCAGGAGAC-3′ were used to amplify GAPDH. All real-time PCR measurements of Exon IV mRNA were done 1 h after stimulation, because our preliminary data showed that the endogenous mRNA transcription reached the peak value at 1 h and remained constant for at least 3 hours.

### Transfection and luciferase assay

Cultured neurons were transfected on 9 DIV by using Lipofectamine 2000 (Invitrogen). Following manufacturer's instruction, 0.1 µg luciferase reporter plasmid (or 0.1 µg Gal4 and 0.1 µg UAS-Luc) and 0.4 µg Renilla-luc plasmid were mixed with Lipofectamine 2000, and added to 0.3 million cells. For some experiments, neurons were also co-transfected with constructs that express regulatory proteins (such as dominant negative protein kinases). Forty-eight hours after transfection (i.e. on 11 DIV), neurons were stimulated by KCl or NMDA for 6 hours. Cells were lysed and the Luciferase activity of the lysate was analyzed with the Dual-Glo Luciferase Assay System (Promega) according to the manufacturer's instruction. The luciferase activity was measured by a Veritas Microplate Luminometer. Firefly luciferase activity was normalized to Renilla luciferase activity. The activity of luciferase reporter plasmids (such as CaRE1, CaRE2, and CaRE3) was compared to that of pGL3-SV40-luciferase. All luciferase assays were done 6 hours after stimulation, because, consistent with previous studies, significant luciferase up-regulation was observed between 4 to 6 hours. Because the transfection efficiency was higher with DIV 9 neurons, all the luciferase experiments were done with DIV 11 neurons (i.e. 2 days after transfection).

### Western blotting

Neurons on 14 DIV were pretreated with inhibitors for 30 minutes, followed by a 20-minute KCl stimulation. Neuron samples were then harvested in SDS lysis buffer (10 mM Tris–HCl buffer, 10% glycerol, 2% SDS, 0.01% bromophenol blue, and 5% β-mercaptoethanol, pH 6.8), and proteins were separated with SDS-PAGE (Invitrogen). Separated proteins were transferred onto a nitrocellulose membrane, incubated with specific primary antibodies (1∶1000 dilution for anti-pERK1/2 and 1∶5000 dilution for anti-β-actin, Cell Signaling, MA), and then incubated with horseradish peroxidase (HRP)-labeled secondary antibodies (1∶ 5000 for both goat anti-rabbit IgG and goat anti-mouse IgG, Thermo). The signal was detected by an enhanced chemiluminescence system (Thermo). The signal intensity was determined by Scion Image software (Scion Corp. Frederick, Maryland). The pERK1/2 level was normalized to β-actin.

### Data analysis

All results were presented as average ± SEM. Student's t-test (unpaired) was used for comparison between two groups. One-way ANOVA and LSD post hoc analysis were used for comparisons among multiple groups. The difference is considered as statistically significant when p<0.05 is reached.

## Results

### Regulation of BDNF exon IV transcription through promoter IV

Previous studies have demonstrated that the route of Ca^2+^ influx matters for the activity-dependent gene transcription of BDNF exon IV. Because co-application of nifedipine and APV ablates KCl-stimulated exon IV transcription, it was concluded that membrane depolarization triggers transcription through L-VGCC and NMDAR. Here, we examined L-VGCC-mediated transcription by co-application of KCl with NMDAR antagonist APV. The NMDAR-mediated transcription was stimulated by co-application of NMDA and L-VGCC antagonist nifedipine. In both cases, we included CNQX to block non-NMDA type glutamate receptors.

**Figure 1:**
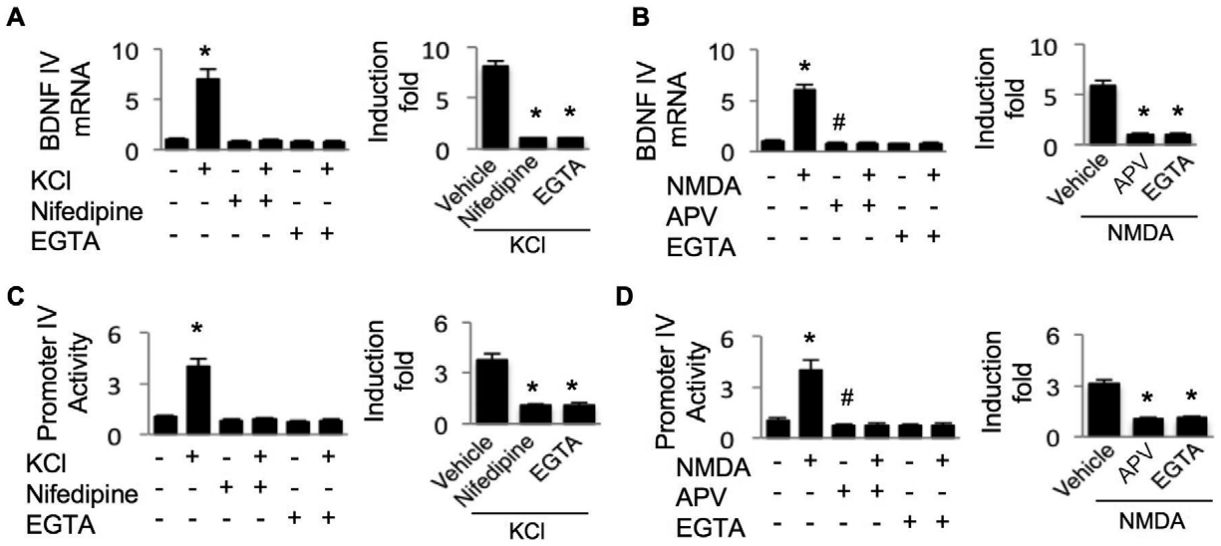
KCl and NMDA stimulate BDNF IV mRNA transcription and PIV-luciferase activity through L-VGCC and NMDAR. For (A) and (B), DIV 14 cortical neurons were treated with 50 mM KCl (A) or 20 µM NMDA (B) for one hour. L-VGCC blocker nifedipine (10 µM), or NMDAR antagonist APV (100 µM), or extracellular calcium chelator EGTA (2.5 mM) was added 30 min before the stimulation. After the one-hour incubation with NMDA or KCl, samples were collected and total RNA was extracted. The extracted RNA was reverse transcribed to cDNA, and then analyzed by real-time PCR. The level of BDNF IV mRNA was normalized to that of GAPDH mRNA. For (C) and (D), DIV 9 cortical neurons were transfected with promoter IV-luciferase reporter and TK-pRL plasmid. Two days after transfection (i.e. on DIV 11), neurons were treated with 50 mM KCl (C) or 20 µM NMDA (D). To pharmacologically block L-VGCC, or NMDAR, or chelate extracellular calcium, neurons were pre-treated with nifedipine, or APV, or EGTA, respectively. After six-hour incubation with KCl or NMDA, dual luciferase assay was performed. The firefly luciferase activity was normalized to Renilla-luciferase. In the left panels, “*” indicates statistical difference to all other groups, and “#” indicates significant difference to the naïve group not treated with any drug. In the right panels, the effect of drugs on KCl- or NMDA-stimulated fold induction of transcription was normalized to the drug-only group (*: statistically different to that of the KCl- or NMDA-stimulated transcription in neurons not treated with nifedipine, or APV, or EGTA). Data were collected from 4 independent preparations for each treatment. All neurons in A and C were pre-treated with CNQX and APV. All neurons in B and D were pre-treated with CNQX and nifedipine.

First, as shown in Figure 1A and 1B, robust up-regulation of exon IV-containing BDNF mRNA was stimulated by KCl or NMDA in DIV 14 cortical neurons. The KCl-induced BDNF IV expression was totally blocked by the L-VGCC antagonist nifedipine (Figure 1A), whereas the NMDA-induced BDNF IV up-regulation was abolished by the NMDAR antagonist APV (Figure 1B). These data indicate that the induction by KCl and NMDA is mediated through the activation of L-VGCC and NMDAR, respectively. Second, the L-VGCC- and NMDAR-mediated increase of BDNF IV mRNA was abolished by inclusion of calcium chelator EGTA in the culture medium, demonstrating the role of extracellular calcium and calcium influx (Figure 1A and B). We also found that the spontaneous activity of NMDAR but not L-VGCC might be involved in maintaining the basal transcription of BDNF IV in non-stimulated neurons. As shown in Figure 1B, in neurons pre-treated with CNQX and nifedipine, blocking NMDAR with APV significantly reduced the level of exon IV-containing mRNA (p<0.05). In contrast, blocking L-VGCC with nifedipine only slightly (without any statistical significance) reduced exon IV (Figure 1A).

**Figure 2:**
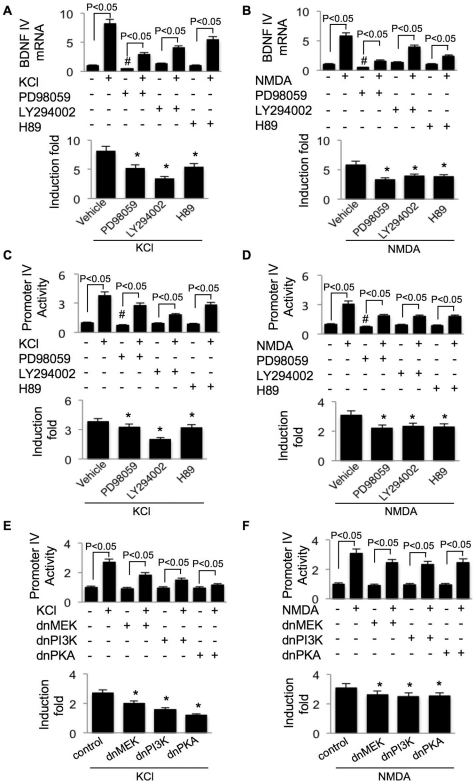
Promoter IV activity correlates with the transcription profile of exon IV-containing BDNF mRNA. For (A) and (B), DIV 14 cortical neurons were stimulated by 50 mM KCl (A) or 20 µM NMDA (B). To block the activity of MEK, PI3K, and PKA, neurons were pre-treated with their inhibitors (i.e. PD98059, LY294002, and H89, respectively) for 30 min before stimulation. After the one-hour incubation with KCl or NMDA, neuron samples were collected and total RNA was extracted. The extracted RNA was reverse transcribed to cDNA, and then analyzed by real-time PCR. The level of BDNF IV mRNA was normalized to that of GAPDH mRNA. From C to F, DIV 9 cortical neurons were transfected with promoter IV-luciferase reporter plus TK-pRL (C and D), or co-transfected with promoter IV-luciferase/TK-pRL and dominant negative constructs of MEK, PI3K, and PKA (E and F). Two days after the transfection (i.e. on DIV 11), neurons were treated with 50 mM KCl (C and E) or 20 µM NMDA (D and F). To pharmacologically block MEK, PI3K, and PKA, neurons were pre-treated with their inhibitors (i.e. PD98059, LY294002, and H89, respectively) for 30 min before stimulation (C and D). After the six-hour incubation with KCl or NMDA, dual luciferase assay was performed. The firefly luciferase activity was normalized to Renilla-luciferase. In the upper panels, “#” indicates significant difference to the naïve group not treated with any inhibitor or stimulator. In the lower panels, the effect of protein kinase inhibitors or dn constructs on KCl- or NMDA-stimulated transcription was normalized to the inhibitor-only or dn construct-only group (*: statistically different to that of the KCl- or NMDA-stimulated transcription in neurons not treated with any protein kinase inhibitor or dn construct). Data were collected from 3 (for A and B) or 8 (from C to F) independent preparations for each treatment.

By using pharmacological inhibitors, we blocked several Ca^2+^-stimulated protein kinases, which have been implicated to regulate CREB activity. Inhibition of MEK (by PD98059) suppressed both basal and L-VGCC- or NMDAR-mediated transcription (Figure 2A and 2B, upper panels). After normalization to the exon IV mRNA level in neurons treated only with PD98059, KCl or NMDA (in the presence of PD98059) stimulated significantly less transcription of exon IV (Figure 2A and 2B, lower panels). Although KCl and NMDA stimulated significant exon IV transcription in the presence of PI3K and PKA inhibitor (Figure 2A and 2B, upper panels), the induction fold of exon IV mRNA increase was significantly less than that in neurons not treated with these inhibitors (Figure 2A and 2B, lower panels). Our result suggests that the MEK-ERK1/2 signaling may also be required for the promoter IV-mediated transcription driven by spontaneous activity in resting neurons. The effects of Ca^2+^ influx and the inhibitors on the mRNA level of exon IV were mainly due to transcription rather than altering mRNA stability, because there was no significant changes in neurons treated with transcription inhibitor actinomycin D (ACD) (Supplemental Figure S1A and S1B).

Cloning of the 911 bp 5′ flanking sequence of exon IV showed that this region is responsible for the Ca^2+^-stimulated promoter activity [21]. Here, we sought for further evidence that this flanking sequence is a *bona fide* promoter for exon IV. We found that the luciferase activity driven by promoter IV (PIV) was stimulated by both L-VGCC and NMDAR in a calcium-dependent manner (Figure 1C and 1D). The level of PIV activity in non-stimulated neurons was affected by APV but not by nifedipine, consistent with what was observed for exon IV mRNA (as shown in Figure 1A and B). Further, pharmacological inhibition of PI3K and PKA suppressed PIV activity in both KCl- and NMDA-stimulated neurons without affecting the basal level (Figure 2C and 2D). In agreement with the function of MEK-ERK1/2 in regulating basal exon IV mRNA level, acute treatment with PD98059 also suppressed PIV-luciferase activity in non-stimulated neurons (Figure 2C and 2D, upper panels). After normalization to the luciferase activity in PD98059-treated neurons, KCl or NMDA (in the presence of PD98059) stimulated significantly less PIV-driven luciferase expression (Figure 2C and 2D, lower panels).

We next tested the function of these Ca^2+^-regulated protein kinases by co-transfecting their dominant negative (dn) constructs with PIV-luciferase. We found that dnMEK, dnPI3K, and dnPKA suppressed the up-regulation of PIV activity in both KCl- and NMDA-stimulated neurons without significantly changing its basal level activity (Figure 2E and 2F). Because the MEK activity was suppressed for about 48 hours in neurons transfected with dnMEK, there might be some compensatory effects to counteract its regulation of the basal PIV activity.

**Figure 3:**
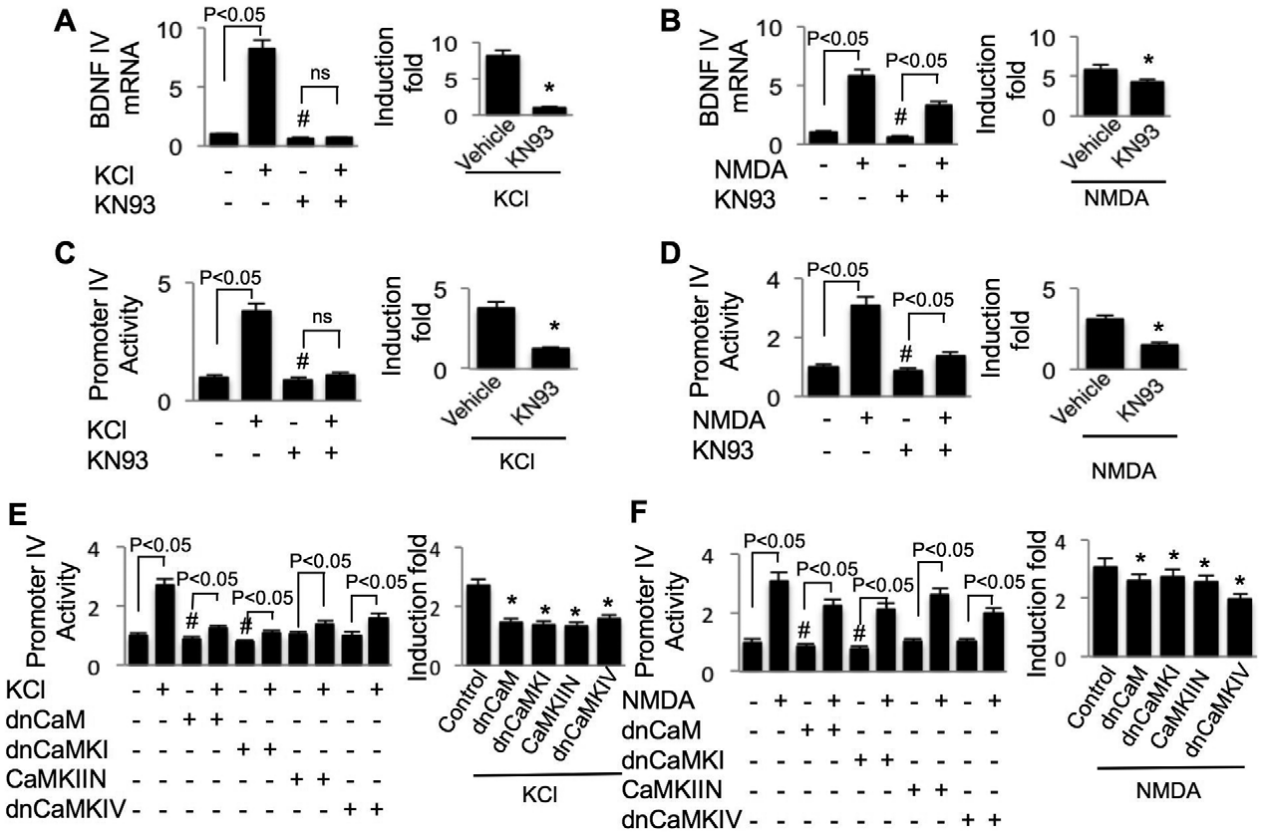
Promoter IV activity is regulated by calmodulin (CaM)-dependent protein kinases I, II, and IV (CaMKI, II, and IV). For (A) and (B), DIV 14 neurons were treated with KN93 for 30 min before stimulation (50 mM KCl in A or 20 µM NMDA in B) to block the activity of CaMKI, II, and IV. Real-time PCR was performed to measure BDNF IV mRNA, which is normalized to GAPDH. For (C) to (F), DIV 9 cortical neurons were transfected with promoter IV-luciferase reporter plus TK-pRL (C and D) or co-transfected with promoter IV-luciferase/TK-pRL and dominant negative construct of CaM, CaMKI, CaMKIV, and CaMKIIN (E and F). Two days after the transfection (i.e. on DIV 11), neurons were treated with 50 mM KCl (C and E) or 20 µM NMDA (D and F). In C and D, KN93 was applied 30 min before stimulation. After the six-hour incubation with KCl or NMDA, dual luciferase assay was performed. The firefly luciferase activity was normalized to Renilla-luciferase. In the left panels, “#” indicates significant difference between the inhibitor/dn construct group and the naïve group not treated with any drug or the dn construct; ns indicates no statistical difference. In the right panels, the effect of protein kinase inhibitors or dn constructs on KCl- or NMDA-stimulated transcription was normalized to the inhibitor-only or dn construct-only group (*: statistically different to that of the KCl- or NMDA-stimulated transcription in neurons not treated with any protein kinase inhibitor or dn construct). Data were collected from 8 independent preparations for each treatment.

We next examined the function of calmodulin (CaM)-dependent protein kinases. CaMKI, II, and IV, which have been implicated in regulating synaptic plasticity and gene transcription [34], are abundant in the central nervous system. Neurons treated with KN93, which is a robust inhibitor for CaMKI, II, and IV, showed lower basal level of exon IV mRNA (Figure 3A and 3B) and PIV activity (Figure 3C and 3D). In the presence of KN93, KCl failed to stimulate PIV and exon IV transcription (Figure 3A and 3C), and the NMDA-induced stimulation was significantly suppressed (Figure 3B and 3D). Blocking CaM or CaMKI activity by dnCaM or dnCaMKI, respectively, blocked both basal and Ca-stimulated (by KCl or NMDA) PIV activity (Figure 3E and 3F). Overexpression of dn constructs of CaMKII and CaMKIV suppressed both KCl- and NMDA-induced PIV activity without affecting the basal levels (Figure 3E and 3F). Because KN93 did not affect the level of exon IV-containing mRNA in the presence of transcription inhibitor ACD (Supplemental Figure S1A), it suggests that the CaM kinases mainly regulate transcription rather than exon IV mRNA stability. These data demonstrate the function of the major Ca^2+^-stimulated protein kinases in regulating BDNF transcription through the 911 bp 5′ flanking sequence.

### Regulation of CaRE1 and CaRE3 activity by calcium influx through L-VGCC and NMDAR

**Figure 4:**
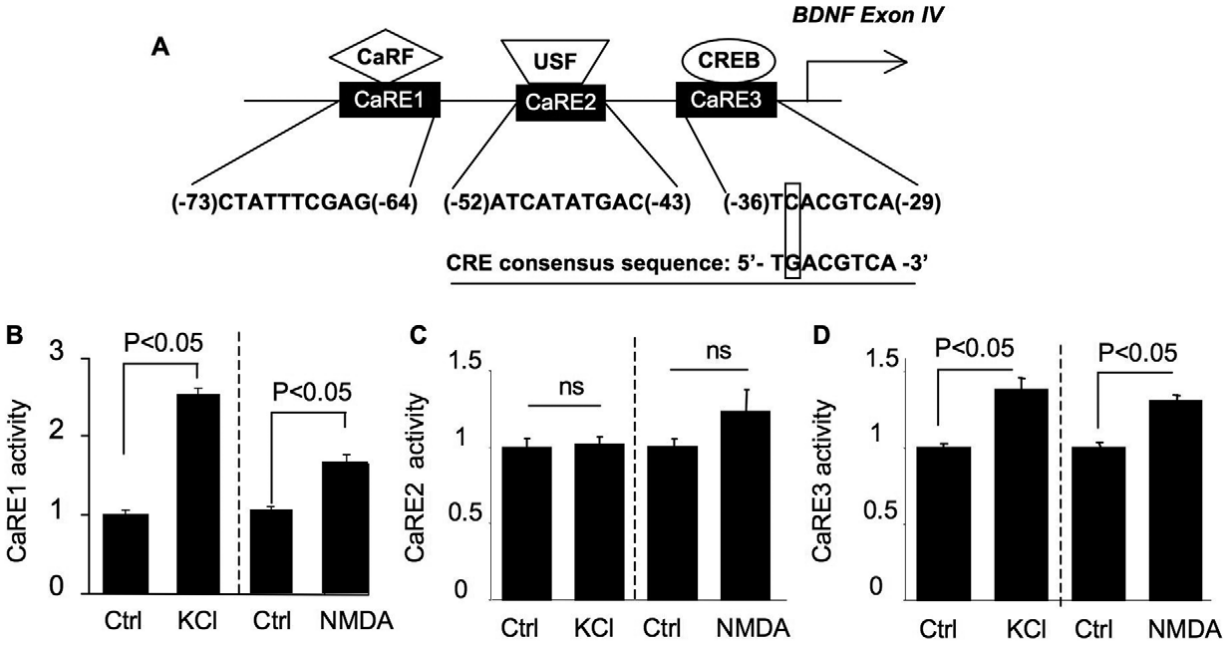
CaRE1 and CaRE3, but not CaRE2 activity, are stimulated by calcium influx. (A) Schematic illustration of the relative positions of CaRE1, 2, and 3 in the promoter IV region of BDNF gene. These calcium responsive elements are binding sites for transcription factors CaRF, USF, and CREB. The nucleotide sequences of CaRE1, 2, 3, and CRE are also shown. From (B) to (D), cortical neurons at DIV 9 were transfected with CaRE1, or 2, or 3-luciferase reporter constructs plus TK-pRL. As a control, the backbone construct (SV40-luciferase) and TK-pRL were co-transfected for normalization purposes. On DIV 11, neurons were treated with 50 mM KCl or 20 µM NMDA. Dual luciferase assay was performed after the six-hour incubation with KCl or NMDA. Neurons used for KCl treatments (both the control and the stimulated groups) were pre-treated with APV and CNQX to block NMDAR and non-NMDA type glutamate receptors, respectively. Neurons used for NMDA treatment (both the control and the stimulated groups) were pre-treated with nifedipine and CNQX to block L-VGCC and non-NMDA type glutamate receptors, respectively. The firefly luciferase activity was normalized to Renilla-luciferase. Then, CaRE-mediated activity was normalized with SV40 activity. SV40 activity was obtained from neurons co-transfected with SV40-luc and TK-pRL. ns: not statistically significant between stimulated and control neurons. For all experiments in this figure, data were collected from 16 independent preparations for each treatment.

Three calcium responsive elements (CaRE) have been identified within the 170 bp 5′ flanking region of exon IV (see Figure 4A). Previous studies have demonstrated that membrane depolarization stimulates CaRE1 activity through L-VGCC [24], [25]. Mutation of CaRE2 and CaRE3 in the context of the 170 bp sequence suppressed KCl-induced transcription. Here, we found that CaRE1-mediated transcription was up-regulated by the activation of both L-VGCC and NMDAR (Figure 4B). For CaRE2, we did not observe any significant stimulation by either L-VGCC or NMDAR (Figure 4C). Next, we found that Ca^2+^ influx through either L-VGCC or NMDAR stimulated CaRE3-mediated transcription (Figure 4D).

### Regulation of CaRE1-mediated transcription by Ca^2+^-stimulated protein kinases

**Figure 5:**
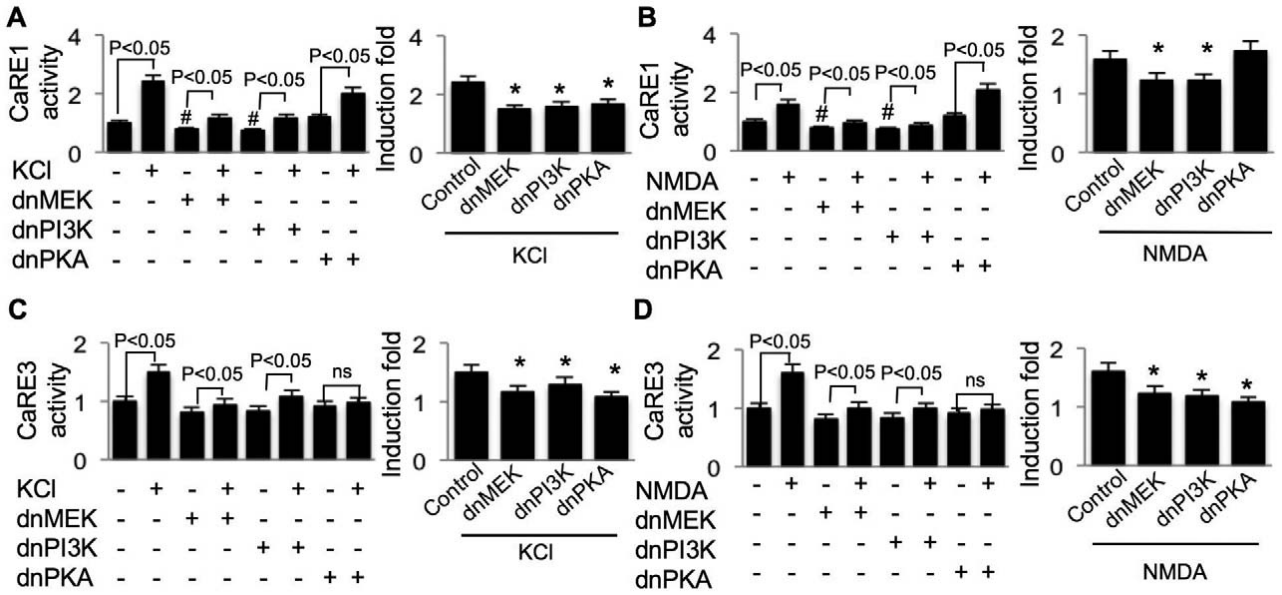
Regulation of Ca^2+^-stimulated CaRE1 and CaRE3 activity by MEK, PI3K, and PKA. DIV 9 cortical neurons were transfected with CaRE1-Luc (A and B) or CaRE3-Luc (C and D) plus TK-pRL and dominant negative MEK, PI3K, and PKA as indicated. For the normalization purpose, SV40-luciferase plus TK-pRL and dominant negative MEK, PI3K and PKA were transfected into different sets of neurons. On DIV 11, neurons were treated with 50 mM KCl (A and C) or 20 µM NMDA (B and D). Dual luciferase assay was performed after the six-hour incubation with KCl or NMDA. After normalized to Renilla luciferase activity, CaRE1- or CaRE-3 activity was further normalized to SV40 activity, and expressed as relative values. In the left panels, “#” indicates significant difference between neurons expressing dn construct and the naïve neurons without dn construct; ns indicates no statistical difference. In the right panels, the effect of dn construct on KCl- or NMDA-stimulated transcription was normalized to the dn construct-only group (*: statistically different to that of the KCl- or NMDA-stimulated transcription in neurons not expressing any dn construct). For all experiments in this figure, data were collected from 12 independent preparations for each experiment.

Although Ca^2+^ influx stimulated significant CaRE1 activity, the regulatory mechanism is unknown. As described earlier, we found that both exon IV transcription and PIV activity were regulated by the major Ca^2+^-regulated protein kinases, including MEK, PI3K, PKA and CaMKs. Here, we sought to determine the regulatory property of the individual CaRE. We found that inhibition of MEK and PI3K (by dnMEK and dnPI3K) suppressed both basal level and KCl- or NMDA-stimulated CaRE1 activity (Figure 5A and 5B). While inhibition of PKA activity by overexpressing dnPKA suppressed KCl-induced up-regulation of CaRE1-mediated transcription without affecting the basal level, PKA activity was not required for NMDA-stimulated CaRE1 up-regulation (Figure 5A and 5B).

**Figure 6:**
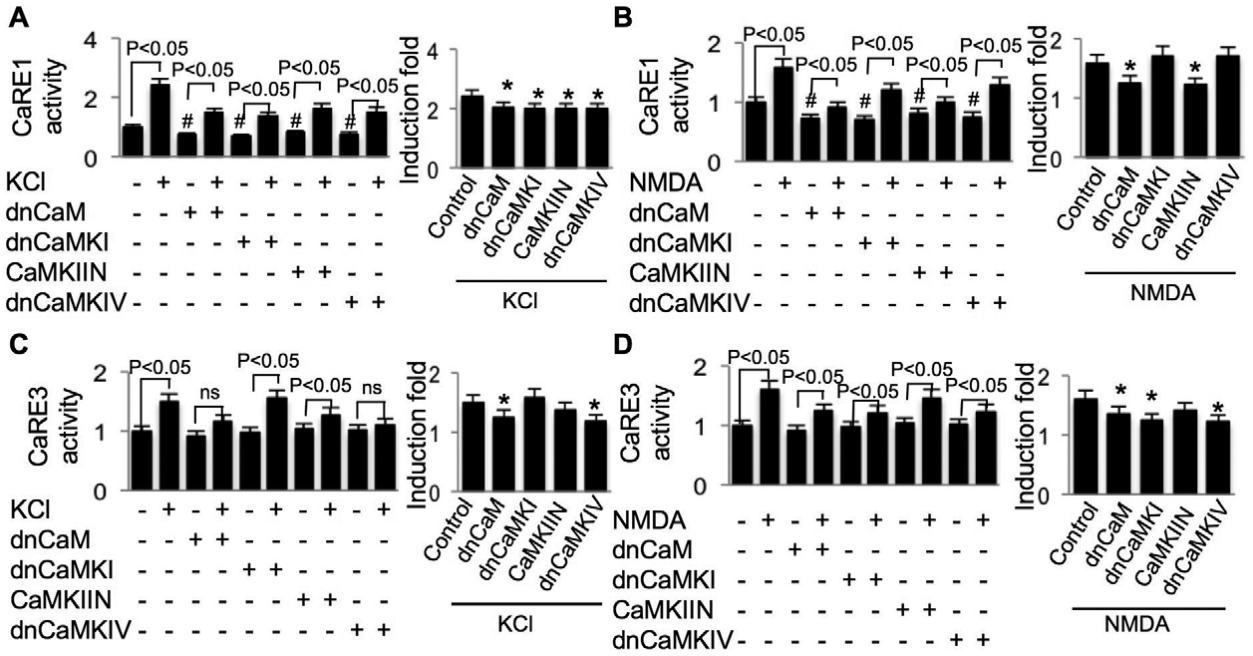
Regulation of Ca2+-stimulated CaRE1 and CaRE3 activity by CaM-dependent kinase I, II, and IV. DIV 9 cortical neurons were transfected with CaRE1-Luc (A and B) or CaRE3-Luc (C and D) plus TK-pRL and dominant negative CaM, CaMKI, CaMKIV, and CaMKIIN. For the normalization purpose, SV40-luciferase plus TK-pRL and dnCaM, dnCaMKI, dnCaMKIV and CaMKIIN were transfected into another set of neurons. Two days after transfection, neurons were treated with 50 mM KCl (A and C) or 20 µM NMDA (B and D), and analyzed by dual luciferase assay after the six-hour incubation with KCl or NMDA. After normalized to Renilla luciferase activity, CaRE1 or CaRE3 activity was further normalized to SV40 activity, and expressed as relative values. In the left panels, “#” indicates significant difference between neurons expressing dn construct and the naïve neurons without dn construct; ns indicates no statistical difference. In the right panels, the effect of dn construct on KCl- or NMDA-stimulated transcription was normalized to the dn construct-only group (*: statistically different to that of the KCl- or NMDA-stimulated transcription in neurons not expressing any dn construct). For all experiments in this figure, data were collected from 12 independent preparations for each treatment.

It has been shown that CaM physically interacts with L-VGCC, and the interaction may be functionally important for signal propagation [35]. As for the function of NMDAR-mediated intracellular signaling, physical interaction between CaMKII and NMDAR may be significant for signaling triggered by NMDAR-mediated Ca^2+^ influx [34]. Here, we found that inhibition of CaM by dnCaM, as well as inhibition of CaMKI, II, and IV by overexpressing their dn constructs, suppressed the basal level of CaRE1 activity in non-stimulated neurons (Figure 6A and 6B, left panels). Although KCl simulated significant up-regulation of CaRE1-mediated transcription in the presence of these dn constructs (Figure 6A, left panel), the induction fold was significantly less (Figure 6A, right panels). The requirement of CaMKI and IV was specific for L-VGCC-mediated CaRE1 up-regulation, because dnCaMKI and dnCaMKIV failed to suppress NMDAR-mediated CaRE1 up-regulation (Figure 6B, right panel). In contrast, inhibition of CaMKII by overexpressing CaMKIIN suppressed both L-VGCC- and NMDAR-mediated CaRE1 up-regulation (Figure 6A and 6B, right panels). These data post an interesting possibility that CaRE1 activity is distinctively coupled to different Ca^2+^ routes (i.e. L-VGCC and NMDAR) via different protein kinases (e.g. PKA, CaMKI, and CaMKIV).

### Regulation of CaRE3-mediated transcription by Ca^2+^-stimulated protein kinases

As shown in Figure 4D, we observed that CaRE3 activity was stimulated by both L-VGCC- and NMDAR-mediated Ca^2+^ influx. We further examined how Ca^2+^-activated protein kinases regulate its activation. We found that inhibition of these Ca^2+^-stimulated protein kinases did not affect the basal activity of CaRE3 in non-stimulated neurons. In KCl-stimulated neurons, overexpression of dnPKA, dnCaM, and dnCaMKIV abolished L-VGCC-mediated activation of CaRE3 (Figure 5C and 6C, left panels). The induction fold of L-VGCC-mediated CaRE3 activation was significantly suppressed by dnMEK and dnPI3K, but not by dnCaMKI and CaMKIIN (Figure 5C and 6C, right panels).

Interestingly, we found that L-VGCC- and NMDAR-mediated activation of CaRE3 was also differentially regulated. Although MEK, PI3K, and PKA activity were required for both KCl- and NMDA-stimulated CaRE3 activity (Figure 5C and 5D), CaM and CaMKs were differentially required. The activity of CaRE3 was stimulated by NMDA but not by KCl in neurons overexpressing dnCaM and dnCaMKIV (Figure 6C and 6D, left panels). The full activation of CaRE3-mediated transcription was suppressed by dnCaMKI and dnCaMKIV but not by CaMKIIN in NMDA-stimulated neurons (Figure 6D, right panel). In contrast, the L-VGCC-mediated activation of CaRE3 required CaMKIV but not CaMKI and CaMKII (Figure 6C, right panel). Consistent with the finding of CaRE1, these data further suggest how Ca^2+^ influx through different routes could exert distinctive effects on the individual CaRE (i.e. both CaRE1 and CaRE3) through different Ca^2+^-activated protein kinases.

### Full Ca^2+^-stimulated activation of CRE and CaRE3 requires the same protein kinases

The DNA sequence of CaRE3 is similar to that of CRE consensus sequence (Figure 4A). Previous results have also demonstrated CREB binding to the CaRE3 sequence [21], [26]. However, there is a possibility that the transcription complex (possibly containing multiple regulators along with CREB) may assemble differently at CaRE3 compared to the classical CRE. Therefore, we decided to further examine whether CRE and CaRE3 are regulated similarly by the major Ca^2+^-activated protein kinases.

**Figure 7:**
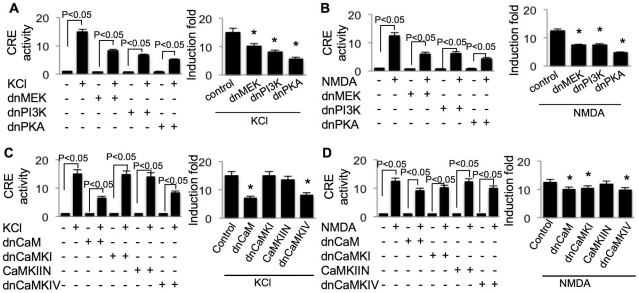
Regulation of CRE by Ca^2+^-stimulated protein kinases. The CRE-luciferase reporter construct plus TK-pRL along with the dominant negative construct of Ca^2+^-stimulated protein kinases (dnMEK, dnPI3K, and dnPKA for A and B; dnCaM, dnCaMKI, CaMKIIN, and dnCaMKIV for C and D) were transfected into cortical neurons on DIV 9. SV40-luciferase plus TK-pRL along with different dominant negative construct were transfected into another set of neurons for normalization purposes. On DIV 11, neurons were treated with 50 mM KCl (A and C) or 20 µM NMDA (B and D). Dual luciferase assay was performed after the six-hour incubation with KCl or NMDA. After normalized to Renilla luciferase activity, CRE activity was further normalized to SV40 activity, and expressed as relative values. In the right panels, the effect of dn construct on KCl- or NMDA-stimulated transcription was normalized to the dn construct-only group (*: statistically different to that of the KCl- or NMDA-stimulated transcription in neurons not expressing any dn construct). Data were collected from 8 independent preparations for each treatment.

Here, we found that the full-scale of Ca^2+^-stimulated up-regulation of CRE required the same set of Ca^2+^-activated protein kinases as those regulating CaRE3. Specifically, both L-VGCC- and NMDAR-mediated CRE activation were suppressed by dnMEK, dnPI3K, and dnPKA (Figure 7A and 7B, right panels). CaM-mediated signaling was also required, because overexpression of dnCaM suppressed L-VGCC- and NMDAR-mediated CRE activation (Figure 7C and 7D, right panels). In KCl-stimulated neurons, overexpression of dnCaMKIV, but not dnCaMKI or CaMKIIN, suppressed CRE-mediated luciferase expression (Figure 7C, right panel). In contrast, the NMDAR-mediated CRE activation was suppressed by dnCaMKI and dnCaMKIV, but not by CaMKIIN (Figure 7D, right panel).

**Figure 8:**
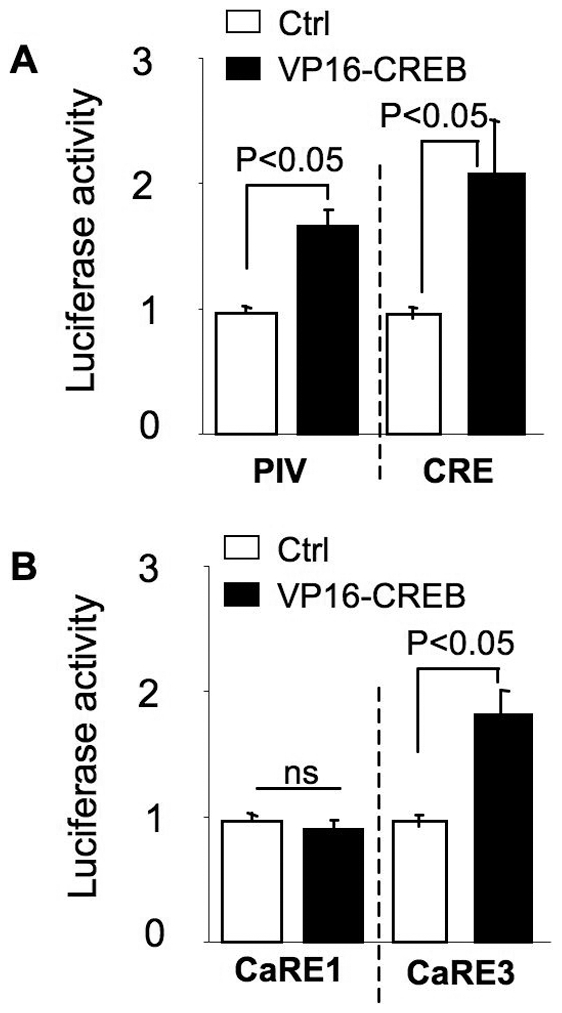
Constitutively active CREB enhances CaRE3 but not CaRE1 activity. (A) Promoter IV- or CRE-luciferase reporter construct was co-transfected with TK-pRL and VP16-CREB expression plasmid into DIV 9 neurons. (B) CaRE1- or CaRE3-luciferase reporter construct was co-transfected with TK-pRL and VP16-CREB expression plasmid into DIV 9 neurons. The activity of promoter IV, CRE, CaRE1, and CaRE3 was determined by dual luciferase assay on DIV 11. After normalized to Renilla luciferase activity, CRE, or CaRE1 or CaRE3 activity was further normalized to SV40 activity in neurons transfected with SV40-luciferase, TK-pRL, and VP16-CREB. ns: not statistically significant. Data were collected from 5 independent preparations for each treatment.

We further tested whether the activation of CREB could stimulate PIV and its calcium responsive elements. As shown in Figure 8A, overexpression of a constitutively active form of CREB (VP16-CREB, which confers transcription factor activity in the absence of upstream CREB activators) significantly enhanced PIV- and CRE-mediated transcription. Consistent with the hypothesis that CaRE3 is a potential CRE, overexpression of VP16-CREB also enhanced CaRE3-mediated transcription (Figure 8B). In contrast, activation of CREB was not sufficient to stimulate CaRE1 activity (Figure 8B).

Although these data demonstrate that both CRE and CaRE3 are regulated by CREB, we found that their regulatory property was not identical. Specifically, KCl stimulated CRE but not CaRE3 activity in neurons expressing dnPKA (Figure 5C and 7A, left panels), dnCaM, and dnCaMKIV (Figure 6C and 7C, left panels). Additionally, inhibition of PKA by dnPKA only abolished CaRE3 activation but allowed partial CRE activation in NMDA-stimulated neurons (Figure 5D and 7B, left panels).

### Regulation of CaRF transcriptional activity by Ca^2+^-stimulated protein kinases

**Figure 9:**
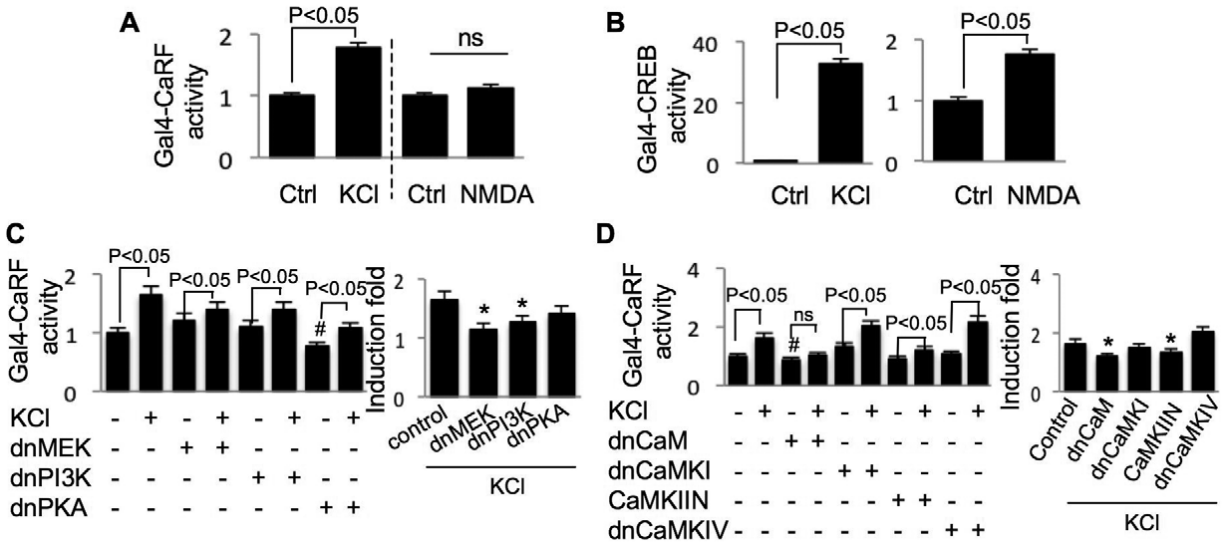
Regulation of CaRF-mediated transcription by Ca^2+^-stimulated protein kinases. Neurons on DIV 9 were transfected with Gal4 only, or Gal4-CaRF, or Gal4-CREB together with Gal4-UAS-Luc and TK-pRL. After 48 hours, the neurons were treated with 50 mM KCl (A) or 20 µM NMDA (B) for 6 hours, and examined by luciferase assay. Dominant negative MEK, PI3K, and PKA (C), or CaM, CaMKI, CaMKIV, and CaMKIIN (D) were co-transfected with the reporter constructs. The data was normalized to TK-Renilla luciferase activity, and then Gal4-CaRF or Gal4-CREB activity was normalized to neurons transfected with Gal4 only. ns: not statistically different. In the left panels of C and D, “#” indicates significant difference between neurons expressing dn construct and the naïve neurons without dn construct. In the right panels of C and D, the effect of dn construct on KCl-stimulated transcription was normalized to the dn construct-only group (*: statistically different to that of KCl-stimulated transcription in neurons not expressing any dn construct). Data were collected from 12 independent preparations for each treatment.

Calcium responsive factor (CaRF) has been identified as a putative transcription factor for CaRE1, because it directly binds CaRE1 *in vitro* [24]. We sought to understand how CaRF-mediated transcription is regulated. We transfected neurons with a plasmid expressing a fusion protein consisting of the DNA binding domain of Gal4 and the transcriptional activation domain of CaRF together with Gal4-UAS-driven luciferase reporter construct. Thus we could measure CaRF transcriptional activity through luciferase assay. Surprisingly, Gal4-CaRF activity was only induced by KCl but not NMDA (Figure 9A). In contrast, Gal4-CREB activity was significantly increased by both KCl and NMDA (Figure 9B). Further, the regulatory property of CaRF was different from that of CaRE1. The L-VGCC-mediated activation of Gal4-CaRF was blocked by dnMEK, dnPI3K, dnCaM, and CaMKIIN (Figure 9C and 9D). Although dnPKA caused significant decrease in basal activity of CaRF (Figure 9C, left panel), the induction fold of KCl-induced activation was intact in neurons expressing dnPKA (Figure 9C, right panel). These data suggest that the regulation of CaRF is different from that of CaRE1. Other mechanisms, such as how binding of CaRF to CaRE1 is regulated, may be involved.

### Involvement of cross-talk between Ca^2+^-stimulated protein kinase pathways

**Figure 10:**
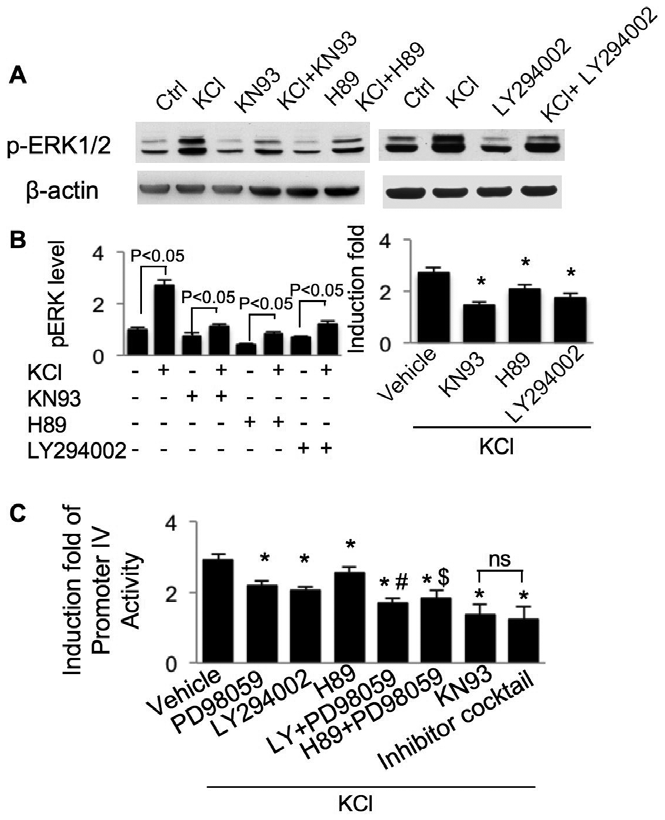
Crosstalk among calcium-stimulated protein kinases and the effects of simultaneous inhibition on promoter IV-mediated transcription. DIV 14 neurons were treated with KN93, or LY294002, or H89 for 30 min before the KCl (50 mM) stimulation. The level of phospho-ERK1/2 and β-actin were examined 20 min after the stimulation by Western blot (A). The signal of pERK1/2 was quantified and normalized to β-actin (B). In the right panel of B, the effects of protein kinase inhibitors on KCl-induced pERK1/2 up-regulation were normalized to the pERK1/2 level in neurons treated with inhibitor only (*: statistically different to that of KCl-stimulated pERK1/2 up-regulation in neurons not treated with protein kinase inhibitors). In C, neurons were transfected with PIV-Luc and TK-pRL on DIV 9. On DIV 11, neurons were first treated with PD98059 (PD), LY294002 (LY), H89, and KN93 individually, or a combination of PD+LY, PD+H89, and PD+LY+KN+H89 (Cocktail) for 30 minutes. Then neurons were stimulated with KCl along with CNQX and APV for 6 hours, and analyzed by luciferase assay. Another set of neurons receiving inhibitor treatment without KCl was used for normalization purposes. The relative up-regulation of PIV activity in neurons treated with KCl+inhibitor was normalized to the PIV-luciferase level in neurons treated with inhibitor only. *: statistically different to that of KCl-stimulated PIV activity in neurons not treated with any inhibitor. #: significant different to KCl-stimulated PIV activity in neurons treated with either PD or LY. $: significant different to KCl-stimulated PIV activity in neurons treated with either H89 or PD. ns: not significantly different. Data were collected from 4 independent preparations for each treatment.

It has been suggested that calcium-sensitive kinases may regulate each other. We focused on MAPK pathway, which has been found to be regulated by other signal transduction pathways [25], [36]. The level of phospho-ERK1/2, which is correlated with ERK1/2 activation, was up-regulated by activation of L-VGCC. Both the basal level and the KCl-induced activation were suppressed by PI3K inhibitor LY294002, PKA inhibitor H89, and CaMK inhibitor KN93 (Figure 10A and 10B). These data confirmed that ERK1/2 activity was significantly disrupted when PI3K, PKA and CaMKs were inhibited.

Because blocking a single pathway showed partial inhibitory effects on PIV-mediated transcription (some inhibitor+stimulator groups still showed significant induction over the corresponding inhibitor-only groups), we tested whether further inhibition could be achieved by simultaneously blocking multiple pathways. Indeed, co-treatment with 2 inhibitors (as indicated in Figure 10C) caused further more inhibition when compared to a single inhibitor. Interestingly, the CaMKs may play a more dominant role than other kinases. As shown in Figure 3A, KN93 totally abolished L-VGCC-stimulated BDNF IV mRNA up-regulation. KN93 also showed more dramatic inhibition of KCl-induced PIV activity (Figure 3C and 10C). Application of more inhibitors (i.e. H89, PD98059, and LY294002) along with KN93 did not cause further inhibition (Figure 10C).

## Discussion

Many aspects of brain function require activity-dependent modifications of gene transcription. Ca^2+^ influx mediated by ligand- and voltage-gated Ca^2+^ channels has been demonstrated as an important molecular trigger for the activation of Ca^2+^-stimulated signaling cascade, which, in turn, turns on gene transcription. BDNF transcription has been studied as a model to delineate how Ca^2+^-stimulated protein kinases regulate the activity of its promoter through the coordination of multiple *cis*-elements and *trans*-factors [21], [24], [25]. The main findings of this study are: 1) the activation of CaRE by Ca^2+^ influx through different routes (L-VGCC and NMDAR) is mechanistically different and involves different Ca^2+^-activated protein kinases, 2) CaRE1 and CaRE3 coordinately mediate BDNF IV transcription, and 3) CaRF shows different regulatory property from CaRE1, and is only sensitive to Ca^2+^ influx through L-VGCC.

It has been shown that Ca^2+^-activated BDNF exon IV transcription is mainly mediated by L-VGCC but not NMDAR [37]. We and others have found that NMDA stimulation can stimulate robust CREB activation and exon IV transcription in younger (e.g. on DIV 3 to 7) but not in mature neurons (e.g. on DIV 14) [22], [38], [39]. Compared to DIV 3–7 neurons, the total expression of NMDAR is higher on DIV 14. Therefore, the same concentration of NMDA (e.g. 50 µM) may cause proper NMDAR activation in DIV 3 neurons, but lead to pathological overactivation and shut off the pro-survival CREB-BDNF signaling in DIV 14 neurons. It was further suggested that deactivation of CREB and BDNF transcription may be mediated by the activation of extrasynaptic NMDARs, because activation of synaptic NMDARs alone always activate CREB and CRE-mediated transcription [40]. It has been shown that lowering the concentration of NMDA significantly stimulates CREB activity and BDNF transcription, and is neural protective [41]. Here, we show that NMDAR-mediated up-regulation of exon IV transcription, as well as PIV, CaRE1, and CaRE3 activity, was significant in mature neurons stimulated with 20 µM NMDA. We also confirmed that the induction by NMDA was through NMDAR and required extracellular calcium.

Although it has been postulated that the property of activity-dependent BDNF IV transcription depends on the route of Ca^2+^ entry [42], [43], how different Ca^2+^ routes dictate different responses remains unclear. This study provides evidence that distinctive Ca^2+^-stimulated protein kinases are differentially required to regulate the activity of the individual CaRE in a Ca^2+^ route-specific manner. It is intriguing that PKA is specifically required for L-VGCC- but not NMDAR-stimulated CaRE1 activation. Previous studies have demonstrated that L-VGCC and NMDAR interact with different intracellular signaling molecules. For example, association of L-VGCC with the PKA anchoring protein AKAP (AKAP15 and AKAP79/150) [44], [45] and CaM may provide a tighter coupling between PKA and CaRE1-mediated transcription.

It has been shown that mutation in CaRE1, 2, and 3 disrupts KCl-induced BDNF IV transcription [21], [24], [25]. We found that, surprisingly, activation of NMDAR or L-VGCC only stimulated CaRE1 and CaRE3 but not CaRE2. It has been reported that CaRE2 is bound by USF1/2, and a consensus USFBE (USF binding element) activity is triggered by membrane depolarization but not by glutamate stimulation [25]. Our results indicate that CaRE2 activity is different from the classical USFBE. Moreover, the previous study stimulated DIV 5 neurons with KCl in the absence of CNQX and APV, whereas we used more mature neurons at DIV 11. We also excluded the involvement of glutamate receptors by co-treatment with CNQX and APV along with KCl. It is possible that CaRE2-mediated transcription is developmentally regulated. Indeed, we were able to detect KCl-mediated CaRE2 activation in DIV 5 neurons (data not shown). Future effort should be made to examine how CaRE2-mediated transcription is regulated in developing neurons.

Studies on the somatostatin promoter have identified a CRE sequence 5′-TGACGTCA-3′, which is stimulated by the elevation of cAMP and PKA activity [46]. Mutation analysis with the BDNF promoter IV has identified a CRE-like sequence 5′-TCACGTCA-3′ (termed as B-CRE or CaRE3) [21], [24], [47]. It has been shown that endogenous CREB binds CaRE3 sequence *in vitro* and *in vivo* [21], [26], [47]. Importantly, genetic mutation of the CaRE3 sequence in mice disrupted activity-dependent up-regulation of BDNF transcription [26]. Although the full-scale activation of CaRE3 and CRE by Ca^2+^ requires the same protein kinases, the difference in nucleotide sequence between them may recruit different transcription complexes. Indeed, this possibility is supported by that inhibition of PKA abolished CaRE3 activation but allowed partial CRE activation by Ca^2+^.

CREB activity could be regulated by multiple Ca^2+^-stimulated protein kinases. It has been demonstrated that phosphorylation of CREB at Ser-133 facilitates the recruitment of coactivator CBP (CREB binding protein) [48], which is critical for CREB-mediated transcription [49]. Activation of CaMKII, CaMKIV and ERK1/2 may all lead to CREB phosphorylation at Ser-133 [27], [50], [51], [52], [53]. Supportively, CaMKIV activity is required for CaRE3/CRE-mediated transcription. However, inhibition of CaMKII has no effect on CaRE3/CRE activity. It may be due to the CaMKII-mediated phosphorylation of CREB at Ser-142, which antagonizes CREB-mediated transcription [53]. Moreover, overexpression of constitutively active CaMKI causes the activation of CREB [53]. Intriguingly, we found that dnCaMKI suppressed NMDAR- but not L-VGCC-mediated CRE and CaRE3 activity. It has been demonstrated that NMDAR-mediated ERK1/2 activation requires the CaMKK-CaMKI cascade [36]. Because the ERK1/2-mediated activation of RSK2 and MSK1 may lead to phosphorylation of CREB at Ser-133 [27], [51], [52], dnCaMKI may suppress CREB activity and CRE/CaRE3-mediated transcription through the ERK1/2-RSK2/MSK1 cascade in NMDA-stimulated neurons.

The crosstalk among Ca^2+^-stimulated protein kinases expands beyond the interaction between CaMKI and ERK1/2. For example, PKA activity may directly regulate Ser-133 phosphorylation of CREB, or indirectly through ERK1/2-RSK2 [54]. On one hand, cAMP elevation and PKA may up-regulate ERK1/2 through RAP [55]. On the other hand, PKA activity is also required for nuclear translocation of ERK1/2 [27]. Further, calcium activation of Ras-ERK1/2 cascade may directly regulate PI3K [56]. Also, PI3K may be up-stream of ERK1/2 activation, as indicated *in vivo* during memory retrieval [28]. During the induction of long-term synaptic potentiation, PI3K also affects high frequency stimulation-induced ERK1/2 activation [57]. Consistent with these previous studies, our data showed that PI3K, PKA, and CaMKs activity impinged on ERK1/2 activation. To add another layer of complication, blocking CaMKs fully abolished the L-VGCC-mediated PIV activation. It is possible that CaMKs affect a broader spectrum of other signaling pathways (in addition to ERK1/2). Alternatively, some of these pathways may work in tandem rather than in parallel to control the sequential steps involved in transcriptional up-regulation.

Because the cAMP-stimulated exon IV transcription is not as robust as Ca^2+^-stimulated transcription, it has been hypothesized that additional *cis*-elements other than CRE may exist in PIV. Indeed, a neurospecific protein CaRF was identified to regulate L-VGCC-mediated CaRE1 activity [24]. Although sequence analysis of CaRF implicates potential phosphorylation target sites of ERK1/2 and CaMKII [24], how these protein kinases regulate CaRE1-mediated transcription and CaRF activity is unknown. Here, we report that Ca^2+^-stimulated CaRE1 activations through L-VGCC or NMDAR are mechanistically different. Our data also showed that CaRE1 activity is independent of CREB. Furthermore, we found that the regulation of CaRF activity was different from CaRE1-mediated transcription. Supportively, a previous study showed that loss of CaRF did not fully phenocopy the mutation of CaRE1 [24], [58], suggesting that additional CaRE1 binding protein may exist. Further, it is possible that there are additional mechanisms to regulate the binding of CaRF to CaRE1.

How do CaRE1 and CaRE3 coordinate to regulate PIV activity? Mutation or deletion of either CaRE1 or CaRE3 ablates Ca^2+^-stimulated up-regulation of PIV activity [21], [24]. This indicates that activation of CaRE1 or CaRE3 alone in the context of flanking sequences of PIV is not sufficient to drive exon IV transcription. Our results are consistent with this possibility, and suggest that coordination and crosstalk between CaRE1 and CaRE3 may be required for the full activity of PIV. This is supported by that blocking either CaRE1 or CaRE3 activity with certain kinase inhibitor (for example, PKA in NMDA-stimulated neurons) inhibits promoter IV activity.

**Figure 11:**
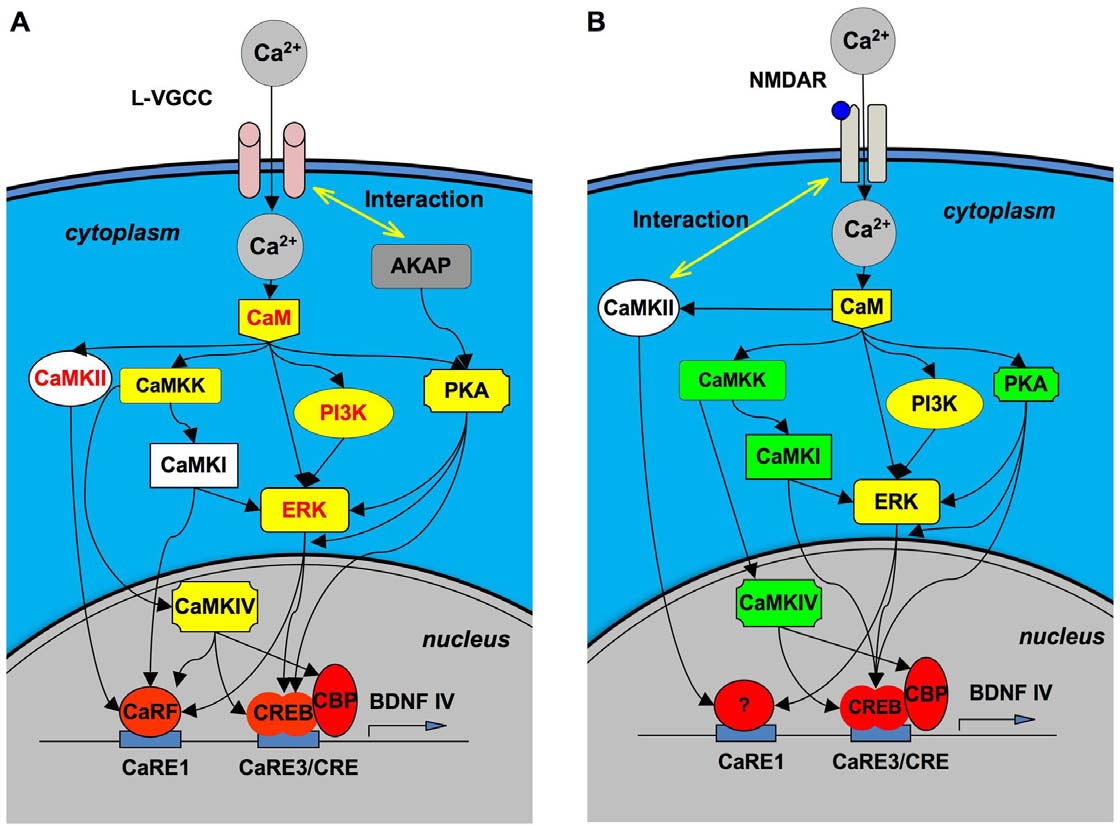
L-VGCC- and NMDAR-mediated activation of CaRE1 and CaRE3/CRE are differentially regulated. Depending on the route of Ca^2+^ entry, different Ca^2+^-stimulated protein kinases are required for L-VGCC- (A) and NMDAR-mediated (B) transcriptional up-regulation of BDNF exon IV. The activation of the major calcium responsive elements, CaRE1 and CaRE3/CRE, also require different signaling molecules. The functional molecules in the Ca^2+^-triggered signaling cascade are connected by arrows pointing from up-stream regulators to down-stream targets. For both (A) and (B), protein kinases in the white boxes are required for CaRE1 activation. Protein kinases in the green boxes are required for CaRE3/CRE activation. Protein kinases in the yellow boxes are required for the activation of both CaRE1 and CaRE3/CRE. Therefore, the changing of box colors for a specific molecule between (A) and (B) indicates the different mechanism underlying L-VGCC- and NMDAR-mediated transcription. For (A), the signaling molecules written in red are those required for CaRF activation. Therefore, based on the color of the box and the color of the word, the regulatory difference between CaRE1 and CaRF could be discriminated. For (B), the “?” indicates that the transcription factors need to be identified for NMDAR-mediated CaRE1 activation.

In summary, we systematically examined the function of the major Ca^2+^-stimulated CREB/CaRF kinases. We found that, depending on the route of calcium entry, CaRE1 and CaRE3 were differentially regulated by different protein kinases. Based on our results and the previous studies, we propose a working model to show how crosstalk between different signaling cascades and coordination of multiple CaREs regulate the activity-dependent transcription of BDNF (Figure 11).

## Supporting Information

Figure S1 **BDNF IV mRNA stability is not affected by KCl/NMDA stimulation or inhibition of the protein kinases.** DIV 14 neurons were incubated with transcription inhibitor actinomycin D (ACD, at 100 ng/ml) before treatment with PD98059, LY294002, H89, KN93, 50 mM KCl, or 20 uM NMDA. Real-time PCR was performed to measure BDNF IV mRNA, which was then normalized to GAPDH. No significance was detected between the treated group and the control group. Data were collected from 8 independent preparations for each treatment.
(TIF)
